# Progress in Medicine

**Published:** 1897-01-09

**Authors:** 


### Progress in Medicine.

### DISEASES OF THE NERVOUS SYSTEM.

(*Continued from page* 233.)

**Hemiatrophy of the Tongue.**—The etiology, pathology, and symptoms of hemiatrophy of the tongue are critically reviewed by C. W. Burr[13] in connection with the report of a case under his care. He considers chiefly those cases due to disease in or near the medulla, excluding the most frequent cases due to direct injury of the hypoglossal nerve by a wound or by pressure of a tumour, and the exceedingly rare cases of peripheral neuritis. He mentions that of late years the condition has been studied very carefully in relation with the affections of the spinal cord, and that its association with locomotor ataxia is relatively so frequent, though absolutely so rare, that in any case in which the course of wasting has been chronic, the possibility of the existence of locomotor ataxia should be thought of. Its absolute rarity in association with posterior sclerosis is shown by the fact that in Koch and Marie's[14] paper only six cases are mentioned. It is a rare complication of general paralysis, Dudley[15] and Ormerod.[16] It may also occur in syringomyelia, one case having occurred in the author's own practice; or may not be associated with any spinal cord disease. Of the latter class of cases Burr cites numerous instances, including cases in which the lesion, either bulbar or peripheral, was syphilitic, or a localised apoplexy, or due to hydatids, caries of vertebræ, phlegmonous inflammation, &c., a list too full and complete to cite here. But, based as they are on a careful consideration of the reports of published cases, we quote in full Burr's conclusions as follows: Hemiatrophy of the tongue with palsy can only be caused by a lesion of the hypoglossal nerve or its nucleus in medulla. Atrophy will not occur if the disease is situated anywhere above the nucleus. The nerve, is purely motor, and has no sensory functions. When there is hemiatrophy without symptoms referable to other nerves, the lesion is almost certainly in the nerve trunk, since the nucleus is in such close relation with other nuclei that no lesion which occurs clinically is at all apt to affect it without involving them. On the other hand, the presence of symptoms referable to other nerves does not prove that the lesion is within the medulla, because the hypoglossal, pneumogastric, and spinal accessory nerves are so close together that a lesion affecting one, as for example, meningitis, is apt to involve all. In cases due to nuclear disease, fibrillary twitching is much more apt to be found than in neuritis, but as appears from a case recently reported by A. Marina,[17] twitching may be present in peripheral disease. The lesions found post-mortem vary greatly. There may be chronic nuclear degeneration, especially in association with diseases of the spinal cord, the results of embolism, thrombosis, or hæmorrhage, a tumour arising within the medulla, and confined to one side, a tumour growing from the membranes or bone, meningitis, caries, hydatid cysts, or direct injury from wound. It is probably more frequently associated with locomotor ataxia than with any other disease of the spinal cord, but it may occur with syringomyelia and in the spinal type of general paralysis. There is no reason why it should not occur in insular sclerosis. In bulbar paralysis the atrophy, like the other symptoms, is practically always bilateral. Most often the patient cannot give a clear account of the beginning of the trouble. If accurate histories could be obtained from patients, it is probable that apoplectiform cases would be found to be more numerous than now appears. The most common clinical picture is hemiatrophy, with greater or less change in the electrical response of the muscles, and with palsy of the palate and larynx on the same side, accompanied by fibrillary twitching. Very often there is persistent head pain in the occiput on the corresponding side, and this seems to be more apt to occur and to be more severe in cases in which the lesion is not within the medulla, and hence is of slight value in making the diagnosis of locality. The accompanying symptoms may be much more widespread than those mentioned above. Thus there may be wasting of the muscles supplied by the spinal portion of the spinal accessory and of the arm, and indeed, as in one case, the hemiatrophy may be only the remainder of a wasting which has affected to some degree all four extremities. If the lesion is within the medulla, since it will be above the crossing of the pyramids, there may be hemiplegia upon the side opposite the hemiatrophy. There may be symptoms referable to the heart and lungs, due to implication of the pneumogastric. The ocular symptoms which sometimes occur, are a complication due either to an independent nuclear lesion or the extension of a meningitis, say, rather than a part of the natural course of the affection.

The most frequent predisposing cause probably is syphilis. It probably will be proven in the future that the poisons of the acute infectious fevers as, for example, scarlet fever, are occasional factors in causation, but thus far a sufficient number of cases have not been studied with regard to this to warrant any positive statement. It is curious that it is not often found in diphtheritic palsy. The influence of lead is still in dispute. The affection may, of course, occur in childhood or old age, but is most frequent in mature life. The diagnosis of the existence of hemiatrophy can be made without any difficulty. It can scarcely be confounded with progressive facial hemiatrophy, in which there is occasionally implication of one-half of the tongue. Acute hemiglossitis needs only to be remembered for the differential diagnosis to be clear. The diagnosis of the immediate cause and the exact pathological condition is never easy, and may be impossible. It can only be made by a study of the accompanying symptoms.

An instance of acute hemiatrophy of the tongue is recorded by Karplus.[18] Certain indications of hereditary syphilis were present. The patient had suffered for three nights from severe headache and pain behind the left ear, which gave rise to temporary disturbance of speech and mastication, and permanent paræsthesia on the left side of the tongue, which deviated to the left, and presented atrophy, fibrillary contractions, and partial R.D.; taste and sensation, not altered—no other cerebral nerves implicated. A similar case is recorded by Marina,[19] which was probably due to an infectious neuritis. Contraction had occurred, in addition to the other usual phenomena; but taste and sensation were normal.

**Acute Pontine Lesions.**—Dana[20] records cases illustrating the very close simulation of opium poisoning by such lesions. A man, aged forty-nine, who a year before the final disease had a number of attacks of palpitation with anginal symptoms, fell from the steps of a train, striking the back of his head. He was dazed, and the next day had apparently an epileptic attack without paralysis. A month later he had a fainting spell, became profoundly unconscious with pin point pupils, respiration one or two a minute, and absent radial pulse. The heart was beating irregularly at about 70; the extremities were cold. With stimulation and artificial heat the respirations increased to four, but soon ceased entirely, very suddenly. After artificial respiration for a quarter of an hour breathing began again, and at the end of five hours became normal. The pupils were normal, and consciousness partially restored. Active delirium then began, lasting three days. After that ceased, and the patient became rational, persistent hiccough came on. There were evidences of arterial degeneration, and weak heart. Had the patient died early in the attack the medical evidence of opium poisoning would have been very strong. Yet this could be positively excluded. Evidently there was acute softening or anæmia of the pons, which simulated opium poisoning just as hæmorrhage of the pons does. (The high temperature of hæmorrhage was absent in this case.) Cases in which the mistake has been made are reported by Wilks, Fagge, and others. In some instances of hæmorrhage the stomach has been washed out and antidotes administered.

**Klumpke's Paralysis.**—Heubner[21] reports three cases of this interesting disease, all in children. In one case there was an osteosarcoma in the region of the seventh cervical and first dorsal vertebræ, with internal metastases, causing atrophy of the corresponding nerves at their exit from the intervertebral foramen. In this case there were the following symptoms: Paralysis and atrophy of the interosseus muscles of the ulnar side of the right hand, with claw-fingers; the electric excitability of the muscles reduced; no reaction of degeneration; the right eyelid and pupil narrowed; the pupil reacted well to light; the lid could be raised voluntarily. There were no vasomotor symptoms. Shortly before death paresis of the lower limbs and retention of urine, with incontinence, came on. The second case was one of caries of the seventh cervical and first dorsal vertebræ. The symptoms were much like those of the first case, but the muscles supplied by the radial nerve were involved more than those innervated by the ulnar, and gave the reaction of degeneration. In the third case there was no autopsy, but the symptoms were like those in the other two. In all three cases there was the paralysis of the arm, with the peculiar ocular symptoms. The latter are explained by the implication in the vertebral disease of the communicating branch of the first dorsal nerve, which, as Klumpke showed experimentally, carries fibres from the cilio-spinal centre to the smooth muscular fibres of the orbit and pupil.

**Spasmodic Torticollis.**—Reports of some interesting cases are given by Pattee,[22] who considers the abnormal contractions the result of irritation of the external cervical branch of the spinal accessory. This complex nerve has its origin in the medulla as well as in the spinal cord, so it is a very difficult matter to locate the exact place of irritation. As it anastomoses with the pneumogastric, a reflex irritation from gastric disturbance may be a cause, or other reflexes may operate. No lesion of the nerve itself has been found. In one case, that of a woman, aged thirty-five, the spasmodic torticollis had existed three years. Nearly all her teeth were more or less decayed, still they had never troubled her very much. All her teeth were extracted, and one grain of sulphate of zinc was given after each meal. In less than one week a great improvement became apparent. In three months she was perfectly well, and no relapse occurred. Pattee considers that here the cause undoubtedly was reflex irritation from the teeth.

Another case was in a man, aged thirty-five. Contraction of the scaleni muscles continued for a year despite various treatment. It had commenced after riding several hours on a hot, dusty day and sneezing a great deal. Hot shower-baths night and morning for fifteen minutes on the neck and shoulders ordered. In six weeks he was perfectly well and there was no relapse.

**Acute Ascending (Landry's) Paralysis.** — Pearce Bailey[23] and Ewing describe a case of acute ascending paralysis, with special reference to its anatomical lesions, and review the cases of Landry's paralysis hitherto recorded, with a view of determining the pathological nature of the disease.

[13] Journ. of Nerv. and Ment. Dis. July, 1896. [14] Rev. de Med., Vol. vii. p. 1. [15] Brain., Vol. xxx. [16] St. Barthol. Hosp. Rep., Vol. xxi. [17] Neurolog. Centralbl., April 15, 1896; Internat. Med. Mag., July, 1896. [18] Wien. Klin. Woch, Apr. 9, 1896; et ibid. [19] Loc. cit. et ibid. [20] The Post Graduate, Vol. xi., No. 7; Amer. Journ. of Med. Sci., Oct., 1896. [21] Charité Annalen, Bd. xx., p. 280; Amer. Journ. of Med. Sci., Oct., 1896. [22] Journ of Amer. Med. Assoc., xxvii., No. 2, p. 59; Amer. Med. Surg. Bullet., Sept. 5, 1896. [23] New York Med. Journ., July 4, 1896.

